# Associations of *GNAS* and *RGS* Gene Polymorphisms with the Risk of Ritodrine-Induced Adverse Events in Korean Women with Preterm Labor: A Cohort Study

**DOI:** 10.3390/pharmaceutics14061220

**Published:** 2022-06-08

**Authors:** Eun-Jeong Jang, Young-Ju Kim, Han-Sung Hwang, Jeong Yee, Hye-Sun Gwak

**Affiliations:** 1 College of Pharmacy and Graduate School of Pharmaceutical Sciences, Ewha Womans University, Seoul 03760, Korea; heimdall01@hanmail.net; 2 Department of Obstetrics and Gynecology, Ewha Womans University School of Medicine, Seoul 07985, Korea; kkyj@ewha.ac.kr; 3 Department of Obstetrics and Gynecology, Konkuk University Medical Center, Konkuk University School of Medicine, Seoul 05030, Korea; hwanghs@kuh.ac.kr

**Keywords:** adverse events, ritodrine, *GNAS*, *RGS2*, *RGS5*, polymorphisms

## Abstract

Ritodrine, a β2-adrenergic receptor agonist, is among most commonly prescribed tocolytic agents. This study aimed to evaluate the associations of single nucleotide polymorphisms in *GNAS, RGS2*, and *RGS5* with the risk of ritodrine-induced adverse events (AEs) and develop a risk scoring system to identify high-risk patients. This is the prospective cohort study conducted at the Ewha Woman’s University Mokdong Hospital between January 2010 and October 2016. Pregnant women were included if they were treated with ritodrine for preterm labor with regular uterine contractions (at least 3 every 10 min) and cervical dilation. A total of 6, 3, and 5 single nucleotide polymorphisms (SNPs) of *GNAS*, *RGS2*, and *RGS5* genes were genotyped and compared in patients with and without ritodrine-induced AEs. A total of 163 patients were included in this study. After adjusting confounders, *GNAS* rs3730168 (per-allele odds ratio (OR): 2.1; 95% confidence interval (95% CI): 1.0–4.3) and *RGS2* rs1152746 (per-allele OR: 2.6, 95% CI: 1.1–6.5) were significantly associated with ritodrine-induced AEs. According to the constructed risk scoring models, patients with 0, 1, 2, 3, 4, and 5 points showed 0%, 13%, 19%, 31%, 46%, and 100% risks of AEs. This study suggested that *GNAS* and *RGS2* polymorphisms could affect the risk of AEs in patients treated with ritodrine.

## 1. Introduction

Preterm birth is defined as delivery before 37 completed weeks of gestation [1]. Since it is one of the major causes of neonatal morbidity and mortality, it has a critical socioeconomic impact on healthcare systems [2]. According to the World Health Organization, about 15 million babies are born premature every year, and 1 million children die because of the complications associated with preterm birth [3]. The main cause of preterm labor is not well established; however, the etiology is considered complex and multifactorial. Many factors have been reported to be associated with increased risk of preterm birth, such as low socioeconomic status, maternal age, multiple gestations, stress, smoking, alcohol or substance abuse, inflammation, and intrauterine infection [4,5].

Various tocolytic agents are used to prevent preterm birth by relaxing uterine contraction via different mechanisms [6]. They include β2-adrenergic receptor agonists, oxytocin antagonists, calcium channel blockers, non-steroidal anti-inflammatory drugs, and magnesium sulfate [7,8]. Among them, ritodrine, a β2-adrenergic receptor agonist, is among the most commonly prescribed tocolytic agents, particularly in Asia and Europe [9,10]. However, due to safety issues, ritodrine has been withdrawn from the market in several countries, including the United States [11]. The adverse events (AEs) of ritodrine include tachycardia, palpitation, tremor, chest pain, and pulmonary edema [6,12]. These cardiopulmonary AEs can be explained by the stimulation of beta-adrenergic receptors by ritodrine with a lack of uterine selectivity [13]. 

For the safe use of ritodrine, it is important to identify the risk factors for AEs. For example, Shigemi et al. demonstrated that advanced maternal age, obesity, and long treatment duration were significant risk factors for ritodrine-induced AEs [14]. Along with clinical factors, several genetic polymorphisms have been reported to increase ritodrine-induced AEs [15,16,17]. Chung et al. showed that *ADRB2* gene polymorphisms were related to ritodrine-induced AEs [15]. Two next-generation sequencing studies also identified *SERPINA7*, *ADRA1A,* and *CPT2* genes as risk factors for ritodrine-induced AEs [16,17].

As the beta-adrenergic receptors are G protein-coupled receptor (GPCR) proteins, several proteins in the GPCR signaling pathway can affect ritodrine responses [18]. As β2-adrenergic receptors are coupled with stimulatory G (Gs) proteins, and regulator of G protein signaling (RGS) proteins inactivate the signals by increasing GTP hydrolysis [19], we herein focused on Gs α subunit gene (*GNAS*) and two RGS genes (*RGS2* and *RGS5*), which are reportedly involved in the cardiovascular system [20]. Therefore, this study aimed to evaluate the associations of single nucleotide polymorphisms (SNPs) in *GNAS*, *RGS2*, and *RGS5* genes with the risk of ritodrine-induced AEs in Korean women with preterm labor. 

## 2. Methods

### 2.1. Study Patients

This prospective cohort study was conducted at the Ewha Woman’s University Mokdong Hospital between January 2010 and October 2016. The study procedure was approved by the Ethics Committee of the Institutional Review Board (IRB number: 217-1-26) and performed in accordance with the ethical standards of the Declaration of Helsinki. All participants provided written informed consent before enrollment.

Eligible patients were pregnant adults (≥18 years old) treated with ritodrine for preterm labor with regular uterine contractions (at least 3 every 10 min) and cervical dilation. Patients were excluded if they were treated with ritodrine for the McDonald procedure, had severe conditions requiring urgent delivery or surgery (e.g., fetal distress, pre-eclampsia, placenta abruption, spontaneous premature membrane, or spontaneous rupture oligohydramnios), or had comorbidities of cardiovascular disease, asthma, and hyperthyroidism. 

Ritodrine (Lavopa^®^; JW Pharmaceutical, Seoul, Korea) was administered by intravenous infusion. The initial dose was 0.05 mg/min, which was increased every 10 min by 0.05 mg/min until adequate response was achieved. Intravenous treatment was discontinued during uterine quiescence. Thereafter, maintenance therapy was continued via an infusion of 0.05 mg/min for 12–48 h.

Data collection was performed by reviewing paper-based and electronic medical records. The following variables were included for data collection: maternal age, gestational age, weight, height, maximum infusion rate, and ritodrine-induced AEs. The presence of ritodrine-induced AEs was defined as dose reduction of ritodrine or change of ritodrine to other medications because of tachycardia (heart rate ≥ 100 bpm), shortness of breath, palpitation, dyspnea, or pulmonary edema.

### 2.2. Genotyping Methods

Genomic DNA was extracted from whole blood samples using QIAamp DNA Blood Mini Kit (QIAGEN, Hilden, Germany). The following single-nucleotide polymorphisms (SNPs) were genotyped using SNaPshot Multiplex Kit (Applied Biosystems, Foster City, CA, USA): *GNAS* (rs12625436 (NC_000020.11: g.58870158G>A), rs13831 (NC_000020.11: g.58900136A>G), rs6128461 (NC_000020.11: g.58902035T>C), rs7121 (NC_000020.11: g.58903752C>T), rs3730168 (NC_000020.11: g.58903884G>A), and rs6026593 (NC_000020.11: g.58904078A>G)), *RGS2* (rs1856840 (NC_000001.11: g.192842157T>C), rs4606 (NC_000001.11: g.192812042C>G), and rs1152746 (NC_000001.11: g.192827775C>T)), and *RGS5* (rs3806366 (NC_000001.11: g.163145531A>G), rs2815276 (NC_000001.11: g.163155478A>G), rs2662776 (NC_000001.11: g.163195239A>G), rs1509018 (NC_000001.11: g.163218794G>C), and rs6698367 (NC_000001.11: g.163226647C>T)). All procedures were performed according to the manufacturer’s instructions. 

### 2.3. Statistical Analyses

Chi-squared test or Fisher’s exact test was performed to compare patients with and without ritodrine-induced AEs. An additive model was applied to analyze the genetic effects on ritodrine-induced AEs. Haplotype analysis was performed using Plink [21]. To identify independent predictors, multivariable logistic regression analysis was performed using factors with *p*-value < 0.05 in the univariate analysis in addition to age and gestational age. Model discrimination was evaluated by area under the receiver operating curve (AUROC) analysis. A risk scoring system was developed based on the multivariable logistic regression model. To calculate the risk score, each coefficient from the multivariable logistic regression model was divided by the smallest one and rounded to the nearest integer. Statistical analysis was performed using SPSS version 20 (IBM, Chicago, IL, USA). A *p*-value of <0.05 was considered statistically significant. 

## 3. Results

Of 236 enrolled patients, 73 patients were excluded by the following reasons: 38 patients did not have enough samples, 15 patients were in a serious condition before arriving to the hospital, 10 patients received ritodrine for the McDonald procedure, and 10 patients had underlying cardiovascular diseases. Accordingly, a total of 163 patients were included in this study. The mean maternal age and gestational age were 31 years (standard deviation (SD): 4 years) and 30 weeks (SD: 4 weeks), respectively. The mean weight and height were 63 kg (SD: 8 kg) and 161 cm (SD: 5 cm), respectively. Table 1 shows the association between baseline characteristics and ritodrine-induced AEs. The height of the patient was statistically significantly associated with AEs, and patients with a height of <160 cm were at a higher risk of developing AEs than others. Similarly, patients weighing <60 kg showed a trend toward higher risk of AEs; however, the association did not reach statistical significance.

The genetic effects on ritodrine-induced AEs are evaluated in Table 2. Among the studied SNPs, rs7121 and rs3730168 of *GNAS* and rs1152746 of *RGS2* were significantly associated with AEs. The odds ratios (ORs) per variant allele for rs7121, rs3730168, and rs1152746 were 0.60 (95% confidence interval (CI): 0.36–0.99), 2.18 (95% CI: 1.62–3.79), and 2.39 (95% CI: 1.05–5.41), respectively. As two SNPs of *GNAS* (rs7121 and rs3730168) showed significant associations with AEs, we constructed haplotypes of them: H1 (C-G in rs7121-rs3730168; 17.6%), H2 (C-A; 26.9%), H3 (T-G; 53.3%), and H4 (T-A; 2.2%). The most frequent haplotype was H3, containing major alleles at every locus, followed by H2, containing risk alleles. The OR per H2 allele was 1.88 (95% CI: 1.08–3.28).

**Figure 1:**
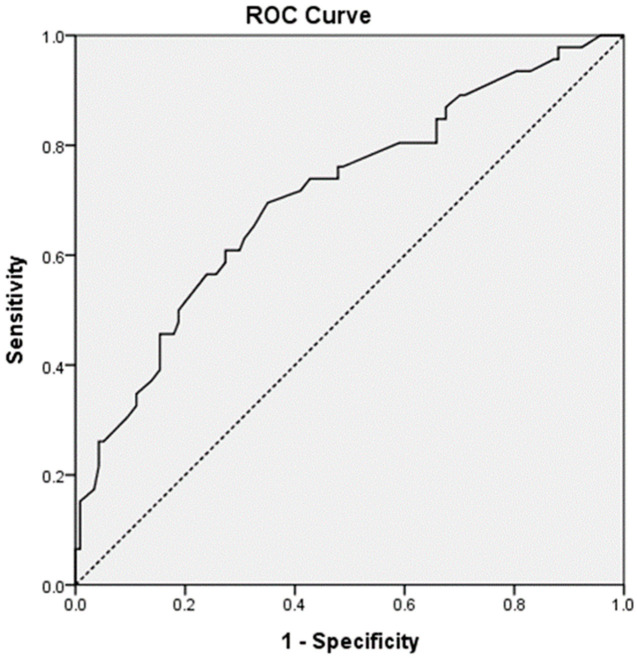
The area under the receiver operating curve for ritodrine-induced adverse events.

Table 3 represents the results of the multivariable logistic regression analysis using age, gestational age, and factors identified as significant (*p*-value < 0.05) in the univariate analysis. After adjusting for confounders, height, *GNAS* rs3730168, and *RGS2* rs1152746 were identified as significant factors related to ritodrine-induced AEs. Patients with a height of <160 cm had a 2.41-fold (95% CI: 1.13–5.12) increased risk of AEs than the others. *GNAS* rs3730168 was associated with a higher risk of AEs (per-allele OR: 2.10, 95% CI: 1.03–4.30). *RGS2* rs1152746 was also associated with a higher risk of AEs (per-allele OR: 2.63; 95% CI: 1.07–6.49). The AUROC for predicted probability by multivariable logistic regression was 0.709 (95% CI: 0.619–0.800, *p*-value < 0.001; Figure 1).

**Figure 2:**
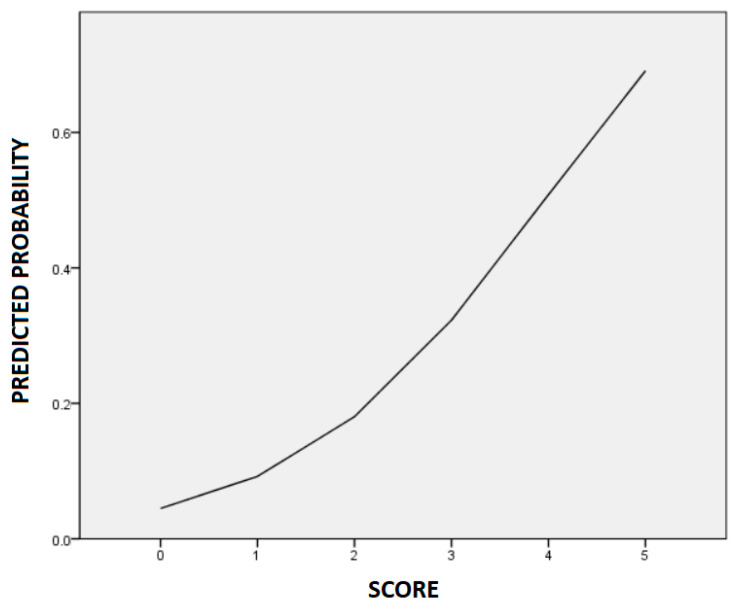
The logistic regression curve of the predicted probability of ritodrine-induced adverse events.

Based on the multivariable analysis model, we constructed a risk scoring system to predict ritodrine-induced AEs. We assigned one point to each factor: height < 160 cm and number of variant alleles (each from 0 to 2) in rs3730168 and rs1152746. Thus, the total risk score range was 0 to 5. According to the constructed risk scoring system, patients with 0, 1, 2, 3, 4, and 5 points were at 0%, 13%, 19%, 31%, 46%, and 100% risks of developing AEs, respectively (Table 4). The risks predicted using the logistic regression curve are shown in Figure 2; the predicted risks of AEs for patients with 0, 1, 2, 3, 4, and 5 points were 4%, 9%, 18%, 32%, 51%, and 69%, respectively.

## 4. Discussion

This study showed that *GNAS* rs3730168 (per-allele OR: 2.10; 95% CI: 1.03–4.30) and *RGS2* rs1152746 (per-allele OR: 2.63; 95% CI: 1.07–6.49) along with height were significantly associated with ritodrine-induced AEs after adjustment for confounders. According to the risk scoring system, patients with higher scores were expected to have higher risks of AEs.

β2-adrenergic agonists have been reported to induce cardiovascular AEs, such as tachycardia, by stimulating β2-adrenergic receptors [22]. In the human heart, β1- and β2-adrenergic receptors are expressed in a ratio of 7:3 and have inotropic and chronotropic effects [23]. Both receptors are coupled to the Gs protein, which stimulates adenylyl cyclase to increase the level of cAMP [24]. It caused cAMP-mediated protein kinase A activation and calcium-induced cardiac contraction, thereby increasing the cardiac output and causing ventricular wall motion [25]. According to studies of mice models overexpressing the Gs α subunit, Gs α overexpression was associated with increased receptor sensitivity and enhanced GPCR signaling pathway, including adenylyl cyclase and L-type calcium channels, thereby leading to increased cardiac contractility and heart rate [26,27,28]. 

RGS proteins are multi-functional GTPase-activating proteins that accelerate the GTPase activity of the G protein α subunit and inhibit GPCR-induced signaling [29,30]. Among several subtypes, RGS2 and RGS5 are predominantly expressed in the cardiovascular system and are known as negative regulators of the GPCR signaling pathway [31]. Several in vivo studies have demonstrated the association of RGS2 inhibition with atrial tachycardia, hypertension, and cardiac hypertrophy [32,33,34]. RGS2 deficiency is reported to prolong vasoconstrictor signaling via AT1R and P2Y and increase internal calcium release via ryanodine receptors [35,36]. Similarly, loss of RGS5 has been reported to be associated with tachyarrhythmia and cardiac hypertrophy in mice [37,38]. Accordingly, genetic variants of *GNAS* and RGS-related genes are hypothesized to affect cardiovascular AEs associated with ritodrine. 

The findings of this study show that rs3730168 of *GNAS* and rs1152746 of *RGS2* are significantly associated with ritodrine-induced AEs after adjustment for confounders. Rs3730168 and rs1152746 are located in the 3’-untranslated region (3’-UTR) of *GNAS* and intronic region of *RGS2*, respectively. Although 3’-UTR and intron are non-coding parts of mRNA, they are known to regulate gene expression. 3’-UTR SNPs may regulate gene expression via degradation, translation, and localization of mRNAs [39], whereas intronic SNPs could affect mRNA stability and alternative splicing [40]. According to the eQTL analysis by GTEx [41], rs3730168 was identified as a significant expression quantitative trait locus, indicating that the variant allele had higher *GNAS* expression in whole blood cells (*p*-value = 8.2 × 10^–6^). Conversely, rs1152746 showed significant eQTL signals in different tissues, mainly with lower expression in the variant alleles. This finding was in agreement with previous reports that suggested that the upregulation of *GNAS* and downregulation of *RGS2* may increase the cardiovascular risk. 

Among the baseline characteristics, height was the only factor associated with ritodrine-induced AEs. As height was correlated with weight (r = 0.366, *p*-value < 0.001) in the study, this can be expected to have high drug concentrations in the smaller patients, thereby leading to AEs. As this was a single-center cohort study, the possibility of biased sampling could not be excluded. Further research is required to confirm these associations. 

## 5. Limitation

This study has several limitations. First, this was a single-center study and included only Asian women, and thus, the findings should be validated in a multicenter study with a larger sample and multiple ethnicities. In addition, we did not perform multiple test corrections to avoid the possible loss of the true positives. 

## 6. Conclusions

This is the first study evaluating the genetic effects of *GNAS*, *RGS2*, and *RGS5* genes on ritodrine-induced AEs in preterm labor women. *GNAS* rs3730168 and RGS2 rs1152746 were identified as risk variants for ritodrine-induced AEs. In addition, we constructed a risk scoring system for predicting ritodrine-induced AEs by incorporating clinical and genetic factors. This tool can be used to identify high-risk patients requiring more caution during ritodrine treatment. 

## Figures and Tables

**Table 1:** Effects of demographic characteristics on ritodrine-induced adverse events.

Parameters	AE	No AE	*p*-Value
Age (years)			0.429
<35	37 (80.4)	100 (85.5)	
≥35	9 (19.6)	17 (14.5)	
Gestational age (weeks)			0.322
<30	24 (52.2)	51 (43.6)	
≥30	22 (47.8)	66 (56.4)	
Weight (kg)			0.057
<60	24 (52.2)	42 (35.9)	
≥60	22 (47.8)	75 (64.1)	
Height (cm)			0.009
<160	22 (47.8)	31 (26.5)	
≥160	24 (52.2)	86 (73.5)	
Maximum infusion rate (cc/hr)			0.799
<60	21 (45.7)	56 (47.9)	
≥60	25 (54.3)	61 (52.1)	

AE: adverse event.

**Table 2:** Effects of genotypes on ritodrine-induced adverse events.

Gene and SNP	Chromosomal Location	Allele A; Allele B ^a^	AE (AA/AB/BB)	No AE (AA/AB/BB)	Odds Ratio (95% CI)	*p*-Value
*GNAS*						
rs12625436	Chr20:58870158	G *; A	7/27/12	23/56/38	0.96 (0.59–1.57)	0.871
rs13831	Chr20:58900136	A *; G	6/25/15	12/50/55	0.68 (0.41–1.13)	0.138
rs6128461	Chr20:58902035	T *; C	8/24/14	19/49/49	0.78 (0.48–1.26)	0.309
rs7121	Chr20:58903752	C *; T	13/23/10	18/60/39	0.60 (0.36–0.99)	0.044
rs3730168	Chr20:58903884	G; A *	17/21/8	63/50/4	2.18 (1.26–3.79)	0.006
rs6026593	Chr20:58904078	A; G *	36/9/1	81/32/3	0.70 (0.34–1.43)	0.322
*RGS2*						
rs1856840	Chr1:192842157	T; C *	15/25/6	47/57/13	1.25 (0.74–2.10)	0.405
rs4606	Chr1:192812042	C *; G	10/30/6	30/59/27	0.87 (0.52–1.46)	0.601
rs1152746	Chr1:192827775	C *; T	0/8/38	3/36/78	2.39 (1.05–5.41)	0.038
*RGS5*						
rs3806366	Chr1:163145531	A; G *	22/18/6	59/49/9	1.20 (0.72–2.02)	0.484
rs2815276	Chr1:163155478	A*; G	8/19/19	14/66/37	1.10 (0.66–1.86)	0.711
rs2662776	Chr1:163195239	A; G *	23/20/3	65/45/7	1.18 (0.68–2.04)	0.566
rs1509018	Chr1:163218794	G; C *	22/18/5	52/54/11	0.94 (0.553–1.59)	0.812
rs6698367	Chr1:163226647	C; T *	29/17/0	58/53/6	0.54 (0.28–1.03)	0.063

AE: adverse event; SNP: single-nucleotide polymorphism. ^a^ Allele A is the reference allele, and allele B is the alternate allele. * Minor allele.

**Table 3:** Multivariable analysis and risk scoring system for ritodrine-induced adverse events.

Parameters	Crude Odds Ratio	Adjusted Odds Ratio	Score
Age ≥ 35 years	1.43 (0.59–3.49)	1.90 (0.69–5.27)	
Gestational age < 30 weeks	1.41 (0.71–2.80)	1.66 (0.79–3.49)	
Height < 160 cm	2.54 (1.25–5.17) *	2.41 (1.13–5.12) *	0, 1 ^a^
*GNAS* rs7121	0.60 (0.36–0.99) *	1.00 (0.51–1.97)	
*GNAS* rs3730168	2.18 (1.26–3.79) **	2.10 (1.03–4.30) *	0, 1, 2 ^b^
*RGS2* rs1152746	2.39 (1.05–5.41) *	2.63 (1.07–6.49) *	0, 1, 2 ^b^

Logistic regression analysis was carried out with variables of age, gestational age, height, *GNAS* rs7121, *GNAS* rs3730168, and *RGS2* rs1152746. * *p*-value < 0.05, ** *p*-value < 0.01. ^a^ Height < 160 cm was assigned to score 1 (0: ≥160 cm; 1: <160 cm). ^b^ Number of the variant allele was assigned to each score (0: GG, 1: GA, 2: AA for rs3730168; 0: CC, 1: CT, 2: TT for rs1152746).

**Table 4:** Observed risks of ritodrine-induced adverse events according to risk scores.

Risk Score	AE	No AE
0	0 (0.0)	2 (100.0)
1	2 (13.3)	13 (86.7)
2	12 (18.5)	53 (81.5)
3	15 (30.6)	34 (69.4)
4	13 (46.4)	15 (53.6)
5	4 (100.0)	0 (0.0)

AE: adverse event.

## Data Availability

The data that support the findings of this study are available from the corresponding author upon reasonable request.
